# Association Between Metabolic Dysfunction-Associated Steatotic Liver Disease and Risk of Aortic Aneurysm and Dissection: A Nationwide Cohort Study

**DOI:** 10.3390/jcm15145453

**Published:** 2026-07-12

**Authors:** Na Kyung Ha, Sang Seok Jeong

**Affiliations:** 1 Dong-A University College of Medicine, Busan 49201, Republic of Korea; hascom333@gmail.com; 2 Department of Thoracic and Cardiovascular Surgery, Dong-A University Hospital, Dong-A University College of Medicine, Busan 49201, Republic of Korea

**Keywords:** metabolic dysfunction-associated steatotic liver disease, MASLD, aortic aneurysm, aortic dissection, cardiometabolic risk factors, Korean National Health Insurance Service

## Abstract

**Background/Objectives**: Prior studies linking fatty liver-related phenotypes to aortic disease have mainly focused on abdominal aortic aneurysm and used earlier nomenclature. We examined associations of metabolic dysfunction-associated steatotic liver disease (MASLD) and cardiometabolic risk factor (CMRF) count with aortic aneurysm and aortic dissection. **Methods:** We analyzed 240,074 adults from the 2009–2010 Korean National Health Insurance Service health-screening database after exclusions. Steatotic liver disease was assessed using the Fatty Liver Index, and MASLD was defined as steatotic liver disease with at least one CMRF. Outcomes were identified using claims-based International Classification of Diseases, 10th Revision codes. Adjusted hazard ratios (HRs) and 95% confidence intervals (CIs) were estimated using Cox models. **Results:** Over a median follow-up of 9.6 years, 1018 aortic aneurysm events and 285 aortic dissection events were recorded. Incidence rates in the MASLD and reference groups were 0.54 versus 0.21 for aortic aneurysm and 0.15 versus 0.04 for aortic dissection per 1000 person-years. Compared with individuals without steatotic liver disease and without CMRFs, MASLD was associated with higher risks of aortic aneurysm (adjusted HR, 1.58; 95% CI, 1.14–2.18) and aortic dissection (adjusted HR, 2.12; 95% CI, 1.03–4.38). Compared with individuals without steatotic liver disease but with CMRFs, MASLD remained associated with aortic aneurysm, but not aortic dissection. Higher CMRF count was associated with aortic aneurysm. **Conclusions:** MASLD and higher CMRF count were associated with incident aortic aneurysm, whereas findings for aortic dissection were less consistent. Further studies with more precise liver and aortic phenotyping are warranted.

## 1. Introduction

Metabolic dysfunction-associated steatotic liver disease (MASLD) was adopted to replace non-alcoholic fatty liver disease (NAFLD) in the 2023 multisociety Delphi consensus to address the exclusionary framing and potentially stigmatizing terminology of the previous nomenclature. MASLD is defined by hepatic steatosis accompanied by at least one cardiometabolic risk factor (CMRF), thereby incorporating metabolic dysfunction into the disease definition [1]. MASLD is highly prevalent worldwide, affecting approximately 30–40% of adults, and is increasingly recognized as a systemic cardiometabolic condition associated with extrahepatic complications, particularly cardiovascular disease [2,3,4]. These observations highlight the need to evaluate MASLD in relation to clinically important aortic disease.

Aortic aneurysm and aortic dissection are relatively uncommon but potentially fatal diseases with substantial clinical burden [5]. Established risk factors for aortic disease include older age, male sex, smoking, hypertension, and family history [6]. Recent evidence suggests that cardiometabolic dysfunction is related to aortic disease, with studies reporting associations of metabolic syndrome and metabolic profiles with aortic aneurysm or dissection [7,8]. However, prior evidence regarding fatty liver-related phenotypes has been derived mainly from studies of abdominal aortic aneurysm (AAA) [9,10], whereas evidence for incident aortic dissection remains limited. Because aneurysm and dissection are related but distinct aortic outcomes, both outcomes should be considered separately when evaluating the association between MASLD and aortic disease.

Despite these prior findings, evidence remains limited regarding MASLD as defined under the updated steatotic liver disease nomenclature, particularly in relation to incident aortic aneurysm and aortic dissection evaluated separately. In addition, because CMRFs are central to the definition of MASLD and the accumulation of metabolic syndrome components has been associated with aortic aneurysm risk [7], evaluating risk according to CMRF count may provide insight into the contribution of cardiometabolic risk accumulation. Therefore, using a nationwide cohort from the Korean National Health Insurance Service, we examined the associations of MASLD with incident aortic aneurysm and aortic dissection separately and evaluated whether risk differed according to CMRF count. We hypothesized that MASLD would be associated with higher risks of these aortic outcomes.

## 2. Materials and Methods

### 2.1. Data Source

Data were obtained from the Korean National Health Insurance Service (NHIS) health-screening database. The NHIS is a nationwide public insurance system that covers approximately 97% of the Korean population. The NHIS also operates the National Health Screening Program, which provides biennial examinations for insured adults aged ≥40 years at government-designated, quality-controlled centers. The database includes sociodemographic information, inpatient and outpatient claims, medical procedures, diagnoses coded according to the International Classification of Diseases, 10th Revision (ICD-10), and health-screening records, including anthropometric measurements, laboratory findings, and self-reported lifestyle factors. Further details on the NHIS database and the National Health Screening Program have been published previously [11,12].

### 2.2. Study Population

**Figure 1:**
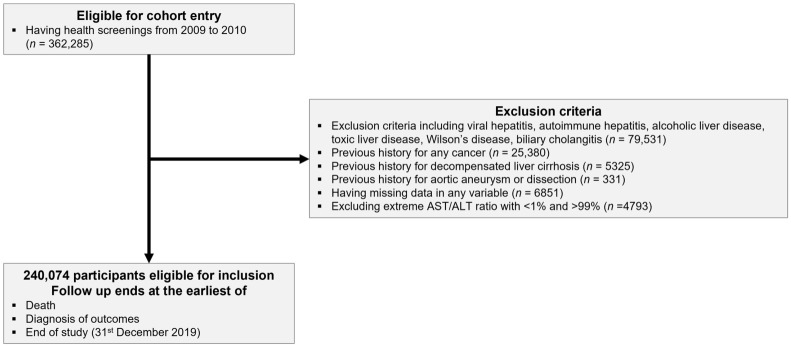
The flow of the study population.

The source population consisted of 362,285 individuals who participated in NHIS health screening during 2009–2010. Diagnostic and exposure classifications were based on International Classification of Diseases, 10th Revision codes and the Fatty Liver Index (FLI) [13,14] (Supplementary Table S2). We excluded participants with liver diseases of distinct etiologies, including viral or autoimmune hepatitis, alcoholic or toxic liver disease, Wilson’s disease, and primary biliary cholangitis (*n* = 79,531). Additional exclusions were applied for previous cancer (*n* = 25,380), decompensated liver cirrhosis (*n* = 5325), prior aortic aneurysm or dissection (*n* = 331), missing data for any variable (*n* = 6851). To account for possible laboratory measurement errors, participants with an aspartate aminotransferase-to-alanine aminotransferase ratio below the 1st percentile or above the 99th percentile were also excluded (*n* = 4793) [15]. The final complete-case analytic cohort included 240,074 participants (Figure 1).

### 2.3. MASLD, CMRFs, Metabolic Dysfunction and Alcohol-Associated Liver Disease (MetALD), and Alcohol-Associated Liver Disease (ALD)

SLD was classified using the FLI, a feasible surrogate measure of hepatic steatosis in large-scale epidemiologic studies [16]. Consistent with international guidelines for population-based research, an FLI score of ≥30 was used to classify SLD [17]. CMRFs were defined according to the five components used in the MASLD diagnostic criteria: (1) body mass index (BMI) ≥ 23 or waist circumference ≥ 90 cm for men and ≥80 cm for women, (2) fasting glucose ≥ 100 mg/dL or current treatment for type 2 diabetes, (3) blood pressure ≥ 130/85 mmHg or use of antihypertensive medication, (4) triglyceride levels ≥ 150 mg/dL or use of lipid-lowering medication, and (5) high-density lipoprotein (HDL) cholesterol < 40 mg/dL for men or <50 mg/dL for women or use of lipid-lowering medication. Participants meeting at least one of these criteria were considered to have CMRFs.

MASLD was defined as SLD with at least one CMRF among participants whose alcohol consumption was below 210 g/week for men and 140 g/week for women. MetALD was defined as MASLD with alcohol consumption of 210–420 g/week in men and 140–350 g/week in women. ALD was defined as SLD with alcohol consumption > 420 g/week for men and >350 g/week for women [1]. Accordingly, participants were categorized into five groups: (1) no SLD without CMRFs, (2) no SLD with at least one CMRF, (3) MASLD, (4) MetALD, and (5) ALD.

### 2.4. Outcomes

Incident aortic aneurysm and incident aortic dissection were evaluated as separate outcomes during follow-up. Incident aortic aneurysm and aortic dissection were identified using claims-based ICD-10 codes in the I71 code family [18]. Aortic dissection was identified using I71.0, and aortic aneurysm was identified using I71.1–I71.9. To improve diagnostic specificity, outcomes were defined as either two or more outpatient claims or one or more hospitalizations with the relevant ICD-10 codes. The index date was defined as the date of the baseline health-screening examination. Participants were followed from the date of health screening in the index year until the first occurrence of incident aortic aneurysm, incident aortic dissection, death, or 31 December 2019.

### 2.5. Covariates

Baseline covariates were obtained from the NHIS database and included demographic characteristics, comorbidities, and health-screening data. Demographic variables consisted of sex, age, household income categorized into quartiles, and residential location classified as urban or rural. For CMRF assessment, hypertension, diabetes, and dyslipidemia were identified using ICD-10 diagnostic codes and relevant prescription records: I10 and I11 for hypertension, E11–E14 for diabetes, and E78 for dyslipidemia, as detailed in Supplementary Table S2. Statins were included within lipid-lowering medication prescriptions for the dyslipidemia definition, whereas statin use, antiplatelet therapy, and anticoagulant therapy were not modeled as separate covariates. Comorbidities were assessed using the Charlson Comorbidity Index and categorized as 0, 1, 2, or ≥3 [19]. Health-screening data comprised BMI, waist circumference, systolic and diastolic blood pressure, fasting blood glucose, total cholesterol, triglycerides, high-density lipoprotein cholesterol, low-density lipoprotein cholesterol, aspartate aminotransferase, alanine aminotransferase, gamma-glutamyl transpeptidase, hemoglobin levels, and estimated glomerular filtration rate. Lifestyle variables were obtained from self-reported questionnaires and included smoking status (never, former, or current smoker), alcohol use (never or current drinker), weekly alcohol intake (g), and frequency of moderate-to-vigorous physical activity (none, 1–2 times/week, 3–4 times/week, or ≥5 times/week).

### 2.6. Statistical Analysis

For baseline characteristics, continuous variables are presented as means (standard deviations), whereas categorical variables are presented as proportions. For comparisons between groups, *t*-tests were used for continuous variables, and chi-square tests were used for categorical variables. Incidence rates were reported separately for aortic aneurysm and aortic dissection as the number of events per 1000 person-years. We used Kaplan–Meier curves to visualize cumulative incidence for both aortic outcomes and log-rank tests to assess differences between groups. Hazard ratios (HRs) and 95% confidence intervals (CIs) for incident aortic aneurysm and incident aortic dissection were estimated using multivariable Cox proportional hazards regression models. The models were adjusted for demographic factors (age, sex, income level, and residential area), health-screening variables (estimated glomerular filtration rate and hemoglobin level), lifestyle factors (smoking status and physical activity), and the Charlson Comorbidity Index [6,14]. Multicollinearity among covariates was assessed using variance inflation factors. All variance inflation factors were below 5, suggesting no substantial multicollinearity among the covariates included in the multivariable model. To assess the proportional hazards assumption, Schoenfeld residuals and graphical inspection of log-minus-log survival plots based on Kaplan–Meier estimates were used. No meaningful violations of the proportional hazards assumption were observed.

To investigate potential nonlinear associations between cardiometabolic risk factors and incident aortic aneurysm and aortic dissection, restricted cubic spline models were fitted with four knots at the 5th, 35th, 65th, and 95th percentiles. Using these models, we evaluated nonlinear dose–response relationships between each aortic outcome and the following continuous cardiometabolic risk factors: BMI, waist circumference, fasting glucose, systolic blood pressure, triglycerides, and HDL cholesterol. To assess the robustness of the findings, sensitivity analyses were conducted using alternative steatosis definitions based on an FLI cutoff of 60 and the Hepatic Steatosis Index (HSI) [13,20]. In addition, competing risk analyses were performed using Fine–Gray subdistribution hazard models, with death treated as a competing event. Subgroup analyses according to sex and age were conducted for incident aortic aneurysm. SAS version 9.4 (SAS Institute Inc, Cary, NC, USA) and R version 4.3.0 (R Foundation for Statistical Computing, Vienna, Austria) were used for all analyses. Statistical significance was set at two-sided *p* < 0.05.

## 3. Results

### 3.1. Baseline Characteristics of the Study Population

At baseline, 79,148 of the 240,074 participants (33%) met the criteria for MASLD. Participants with MASLD were more often male and had higher prevalences of hypertension, diabetes, and dyslipidemia than participants without SLD. The cardiometabolic risk burden was particularly evident in the MASLD group; these individuals had higher BMI and total cholesterol levels, along with lower HDL cholesterol levels. Although MASLD was the primary group of interest, the MetALD and ALD groups showed even more pronounced elevations in systolic and diastolic blood pressure, as well as fasting glucose and triglyceride levels. Regarding liver enzyme profiles, the ALD group had the highest levels of aspartate aminotransferase, alanine aminotransferase, and γ-glutamyl transpeptidase (Table 1). Additional detailed baseline characteristics are provided in Supplementary Table S1.

Supplementary Table S3 presents baseline characteristics according to CMRF count. Hypertension, diabetes, and dyslipidemia became more frequent as the number of CMRFs increased and were most common among participants with all five CMRFs. Furthermore, a greater CMRF burden was associated with an increasingly unfavorable metabolic profile, including higher BMI, systolic and diastolic blood pressure, fasting blood glucose, and triglycerides, along with lower HDL cholesterol.

### 3.2. Association of SLD Status and CMRF Count with Incident Aortic Aneurysm and Aortic Dissection

**Figure 2:**
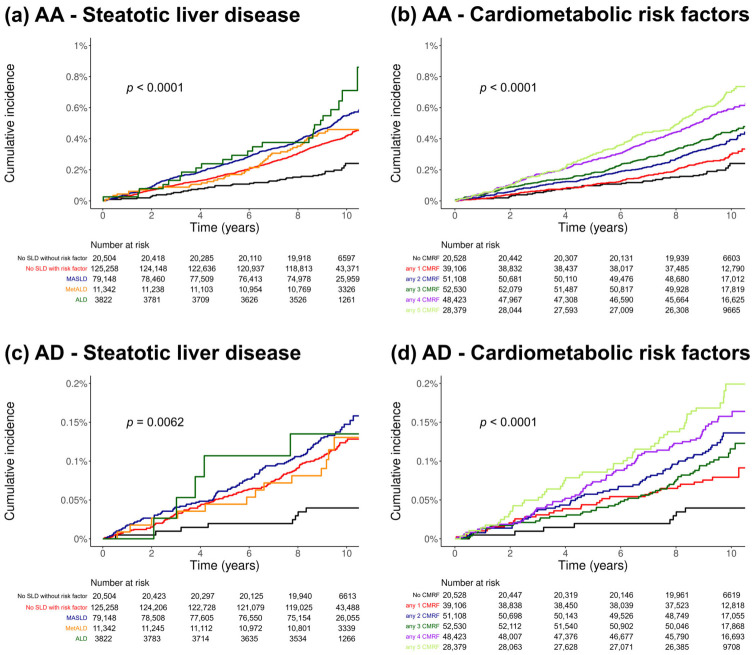
Unadjusted Kaplan–Meier curves for incident aortic aneurysm and aortic dissection according to SLD status and CMRF count. CMRF, cardiometabolic risk factor; MASLD, metabolic dysfunction-associated steatotic liver disease; SLD, steatotic liver disease.

Over a median follow-up of 9.6 years, 1,018 aortic aneurysm events and 285 aortic dissection events occurred. Using participants without SLD and without CMRFs as the reference group, MASLD was significantly associated with a higher risk of both aortic aneurysm (adjusted HR, 1.58; 95% CI, 1.14–2.18; *p* = 0.005) and aortic dissection (adjusted HR, 2.12; 95% CI, 1.03–4.38; *p* = 0.042) (Table 2). The Kaplan–Meier curves in Figure 2 show the cumulative incidence of aortic aneurysm and aortic dissection according to SLD status and CMRF count.

To directly compare MASLD with participants without SLD but with CMRFs, additional analyses were performed using the no SLD with CMRF group as the reference. In this analysis, MASLD was associated with a modestly higher risk of aortic aneurysm after adjustment (adjusted HR, 1.15; 95% CI, 1.01–1.32; *p* = 0.043). However, MASLD was not significantly associated with aortic dissection compared with the no SLD with CMRF group (adjusted HR, 1.05; 95% CI, 0.81–1.35; *p* = 0.720).

When participants were categorized according to CMRF count, a higher CMRF count was associated with an increased risk of aortic aneurysm. Each additional CMRF was associated with a 13% higher risk of aortic aneurysm (adjusted HR, 1.13; 95% CI, 1.08–1.18; *p* < 0.001). For aortic dissection, the per-count association was not statistically significant (adjusted HR, 1.08; 95% CI, 0.99–1.18; *p* = 0.079). Associations for individual CMRF components are shown in Supplementary Table S5. Supplementary Figure S1 provides additional detail on these associations using restricted cubic spline plots, revealing heterogeneous patterns across individual CMRF components and aortic outcomes.

### 3.3. Sensitivity Analyses

We evaluated the robustness of the findings by repeating the main analyses using alternative steatosis definitions, including FLI ≥ 60 and the HSI. Supplementary Table S4 shows that participants with MASLD had a higher risk of aortic aneurysm under both alternative steatosis definitions. When SLD was defined by FLI ≥ 60, the MASLD group had a higher risk of aortic aneurysm after adjustment (adjusted HR, 1.49; 95% CI, 1.04–2.15; *p* = 0.031). Similarly, defining SLD using the HSI yielded an adjusted HR of 1.50 (95% CI, 1.06–2.11; *p* = 0.021) for aortic aneurysm in the MASLD group. For aortic dissection, the associations for MASLD did not reach statistical significance when SLD was defined using FLI ≥ 60 (adjusted HR, 1.74; 95% CI, 0.78–3.89; *p* = 0.180) or the HSI (adjusted HR, 2.06; 95% CI, 0.97–4.37; *p* = 0.059). After adjustment, MetALD and ALD were not significantly associated with either aortic aneurysm or aortic dissection across the alternative steatosis definitions. The HSI-based estimate for ALD and aortic dissection was not available because no events occurred in this group.

Additional competing risk analyses were conducted using Fine–Gray models, with death treated as a competing event. The results were consistent with the primary Cox regression analyses, with MASLD showing significant associations with both aortic aneurysm (HR, 1.61; 95% CI, 1.16–2.23; *p* = 0.004) and aortic dissection (HR, 2.15; 95% CI, 1.04–4.46; *p* = 0.040).

### 3.4. Subgroup Analyses

**Figure 3:**
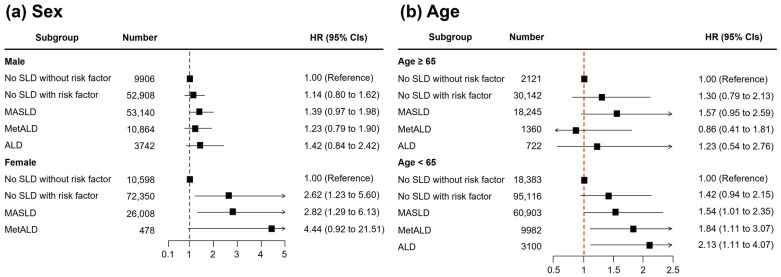
Subgroup analyses of the association between SLD status and incident aortic aneurysm according to sex and age. *p* for interaction was >0.05 for both sex and age. The model was adjusted for age, sex, income level, residence, Charlson comorbidity index, hemoglobin level, glomerular filtration rate, smoking status and physical activity. CI, confidence interval; CMRF, cardiometabolic risk factor; HR, hazard ratio; MASLD, metabolic dysfunction-associated steatotic liver disease; SLD, steatotic liver disease.

We performed exploratory subgroup analyses by sex and age. Because aortic dissection events were limited, these analyses were restricted to incident aortic aneurysm. The *p* values for interaction by sex and age were both >0.05. These subgroup findings are shown in Figure 3.

## 4. Discussion

In this Korean nationwide health-screening cohort, MASLD was associated with higher risks of incident aortic aneurysm and aortic dissection compared with individuals without SLD and without CMRFs. When individuals without SLD but with CMRFs were used as the reference, the association for aortic aneurysm was attenuated but remained statistically significant, whereas that for aortic dissection was not. Additionally, an increasing number of CMRFs showed a progressive association with aortic aneurysm risk, while the per-count risk for aortic dissection did not reach statistical significance. In sensitivity analyses using alternative steatosis definitions, the significant findings were maintained for aortic aneurysm, but the corresponding associations for aortic dissection lost statistical significance. Overall, the association between MASLD and aortic outcomes appeared more consistent for aortic aneurysm than for aortic dissection.

Previous studies have linked fatty liver-related phenotypes, including MAFLD and NAFLD, to AAA development and progression [9,10]. These findings suggest a possible association between hepatic steatosis accompanied by metabolic dysfunction and aortic disease, but prior evidence has largely been based on earlier fatty liver disease nomenclature and has focused mainly on AAA. The present study extends this literature by applying the updated MASLD framework and by evaluating incident aortic aneurysm and aortic dissection as separate outcomes in a nationwide Korean health-screening cohort with long-term follow-up.

The association between increasing CMRF count and aortic aneurysm is consistent with a previous nationwide cohort study showing that AAA risk increased with the accumulation of metabolic syndrome components [7]. In the present study, MASLD was associated with aortic aneurysm when compared with individuals without SLD and without CMRFs; this association was weaker but remained statistically significant in the direct comparison with individuals without SLD but with CMRFs. This suggests that the association between MASLD and aortic aneurysm may not be explained solely by the presence of CMRFs. For aortic dissection, however, MASLD was not significantly associated with risk in the direct comparison, and the per-count association with CMRFs did not reach statistical significance. Still, given the smaller number of aortic dissection events, these dissection-specific findings should be interpreted cautiously. Although the CMRF count analysis supports the relevance of cumulative cardiometabolic burden, the component-specific analyses showed heterogeneous patterns, indicating that cardiometabolic risk accumulation may not be fully captured by a simple additive burden alone. At the component level, all individual CMRF components were associated with aortic aneurysm, whereas only hypertension showed a statistically significant association with aortic dissection. In particular, the insulin resistance component showed an inverse association with aortic aneurysm in the component-specific analysis (Supplementary Table S5). This pattern is consistent with prior studies on diabetes or glycemic status and AAA [21,22], suggesting that the composition of cardiometabolic risk factors may also be important when interpreting cumulative metabolic burden.

Several overlapping metabolic, inflammatory, and vascular-remodeling pathways may help contextualize the observed associations. Previous studies have proposed that fatty liver-related or systemic metabolic dysfunction may be linked to aortic disease through shared CMRFs, oxidative stress, systemic inflammation, immune cell activity, and vascular wall remodeling [4,9]. These pathways overlap with features of aortic wall vulnerability implicated in both aortic aneurysm and aortic dissection, including aortic wall inflammation, extracellular matrix remodeling or degradation, elastin fragmentation, medial degeneration, and vascular smooth muscle cell dysfunction [10,23,24]. Although these mechanisms may provide a plausible biological framework for the observed associations, they were not directly evaluated in the present study; further studies are needed to clarify the mechanistic links between hepatic–metabolic dysfunction and aortic disease.

In this study, MASLD was associated with incident aortic disease in a pattern that was more consistent for aortic aneurysm than for aortic dissection. For aortic aneurysm, MASLD was associated with higher risk compared with individuals without SLD and without CMRFs, and the association remained statistically significant, although attenuated, when MASLD was compared with individuals without SLD but with CMRFs. A higher CMRF count was also associated with a graded increase in aortic aneurysm risk. In addition, the association between MASLD and aortic aneurysm was maintained across alternative steatosis definitions and Fine–Gray competing-risk analyses, supporting the stability of this observation. For aortic dissection, MASLD was associated with risk when compared with individuals without SLD and without CMRFs and in the Fine–Gray analysis; however, the association was not statistically significant when MASLD was compared with individuals without SLD but with CMRFs, in the per-count CMRF analysis, or in analyses using alternative steatosis definitions. Therefore, the aortic dissection results require more cautious interpretation, especially given the smaller number of events. Further studies using imaging-confirmed steatosis, assessment of liver disease severity, refined SLD subtype classification, detailed aortic phenotyping, and external validation are needed to validate and refine these observations.

### Limitations

Several limitations should be considered when interpreting our findings. First, the observational design of this study precludes causal inference, and residual confounding may remain despite multivariable adjustment. Complete-case analysis and exclusion of individuals with extreme aspartate aminotransferase-to-alanine aminotransferase ratios may also have introduced selection bias. Second, SLD was assessed using FLI and HSI rather than imaging-based measures or histologic confirmation. Although these indices have been used as noninvasive surrogate markers of hepatic steatosis in epidemiologic studies [14,25], they include metabolic variables that overlap with the MASLD definition and cardiovascular risk profile. Specifically, FLI incorporates BMI, waist circumference, triglycerides, and gamma-glutamyl transpeptidase, whereas HSI incorporates BMI and diabetes in addition to aminotransferase ratio and sex. Therefore, exposure misclassification and metabolic overlap are possible, and the sensitivity analyses using alternative steatosis definitions do not fully resolve this issue. Liver disease severity, including fibrosis, was also unavailable.

Third, alcohol intake was based on self-reported questionnaire data, which may be affected by underreporting and misclassification. In addition, the MetALD and ALD groups were relatively small, predominantly male, and not consistently significant in sensitivity analyses. Therefore, analyses of these alcohol-related SLD categories should be regarded as secondary and interpreted cautiously. Fourth, incident aortic aneurysm and aortic dissection were identified using claims-based ICD-10 definitions. Although requiring hospitalization or repeated outpatient claims may improve diagnostic specificity, outcome misclassification remains possible, and validation evidence for the full range of I71 codes in the Korean NHIS database is limited. Prior Korean claims-based studies have used ICD-10 codes to define aortic aneurysm and dissection outcomes [18,21], but claims-defined aortic disease outcomes may still be vulnerable to misclassification [26]. Detailed aortic information, including imaging confirmation, aortic location distinguishing between the thoracic and abdominal segments, aneurysm size, rupture status, treatment status, and dissection subtype or extent, was unavailable. Although aortic aneurysm and aortic dissection were analyzed separately, the smaller number of aortic dissection events limited statistical precision, precluded subgroup analyses for this outcome, and may have contributed to the less consistent findings for aortic dissection across comparison groups and sensitivity analyses. Finally, because this study was conducted in a Korean national health-screening cohort, external validation is needed to assess generalizability to other populations and healthcare systems.

## 5. Conclusions

In conclusion, MASLD was associated with a higher incidence of aortic aneurysm in this Korean nationwide health-screening cohort, and this association was maintained across alternative steatosis definitions and competing-risk analyses. A higher CMRF count showed a graded association with aortic aneurysm risk. In contrast, the MASLD–aortic dissection association was not maintained across comparator groups or alternative steatosis definitions; these findings should be interpreted cautiously given the smaller number of events. Further studies with more precise liver and aortic phenotyping are needed to validate these observations.

## Figures and Tables

**Table 1:** Baseline characteristics of the study population.

Variables		No SLD Without CMRF (*n* = 20,504)	No SLD with CMRF (*n* = 125,258)	MASLD (*n* = 79,148)	MetALD (*n* = 11,342)	ALD (*n* = 3822)	*p*-Value
Sex (%)	Male	9906 (48.3)	52,908 (42.2)	53,140 (67.1)	10,864 (95.8)	3742 (97.9)	<0.001
	Female	10,598 (51.7)	72,350 (57.8)	26,008 (32.9)	478 (4.2)	80 (2.1)	
Age (years)	Mean (SD)	55.0 (7.2)	59.0 (8.9)	58.9 (8.5)	55.9 (7.2)	57.4 (8.2)	<0.001
Hypertension (%)		0 (0.0)	53,535 (42.7)	44,565 (56.3)	6243 (55.0)	2176 (56.9)	<0.001
Diabetes (%)		0 (0.0)	13,107 (10.5)	15,298 (19.3)	2221 (19.6)	783 (20.5)	<0.001
Dyslipidemia (%)		0 (0.0)	43,916 (35.1)	41,284 (52.2)	4583 (40.4)	1429 (37.4)	<0.001
Charlson comorbidity index (%)	0	13,376 (65.2)	63,059 (50.3)	36,564 (46.2)	6091 (53.7)	1925 (50.4)	<0.001
	1	5205 (25.4)	34,368 (27.4)	21,404 (27.0)	2985 (26.3)	1047 (27.4)	
	2	1529 (7.5)	15,632 (12.5)	10,819 (13.7)	1311 (11.6)	482 (12.6)	
	≥ 3	394 (1.9)	12,199 (9.7)	10,361 (13.1)	955 (8.4)	368 (9.6)	
Body mass index (kg/m^2^)	Mean (SD)	20.8 (1.5)	22.9 (2.2)	26.1 (2.5)	25.4 (2.5)	25.3 (2.6)	<0.001
Waist circumference (cm)	Mean (SD)	73.2 (5.8)	78.3 (6.3)	88.2 (6.2)	88.0 (6.3)	88.1 (6.7)	<0.001
Systolic blood pressure (mmHg)	Mean (SD)	111.7 (9.4)	124.5 (15.0)	128.7 (14.8)	130.4 (14.5)	131.1 (15.1)	<0.001
Fasting blood glucose (mg/dL)	Mean (SD)	87.8 (7.4)	98.4 (21.6)	105.7 (28.2)	108.5 (29.6)	109.7 (30.0)	<0.001
Total cholesterol (mg/dL)	Mean (SD)	189.6 (25.5)	198.9 (36.8)	206.7 (38.5)	203.2 (36.8)	201.1 (37.9)	<0.001
Triglyceride (mg/dL)	Mean (SD)	81.2 (28.3)	106.7 (48.5)	187.9 (96.4)	201.3 (116.6)	202.9 (121.4)	<0.001
HDL cholesterol (mg/dL)	Mean (SD)	62.2 (19.1)	56.2 (23.1)	50.5 (25.6)	53.5 (20.0)	56.0 (29.7)	<0.001
Alcohol drinking (%)		7763 (37.9)	40,106 (32.0)	35,517 (44.9)	11,342 (100.0)	3822 (100.0)	<0.001
Amount of Alcohol drinking (g/week)	Mean (SD)	41.3 (95.9)	37.0 (96.8)	39.1 (57.1)	287.3 (70.1)	644.7 (262.2)	<0.001
Fatty liver index	Mean (SD)	7.6 (5.5)	15.0 (7.7)	50.9 (15.8)	57.7 (17.7)	60.2 (18.1)	<0.001

ALD, alcohol-associated liver disease; CMRF, cardiometabolic risk factor; HDL, high-density lipoprotein; MASLD, metabolic dysfunction-associated steatotic liver disease; MetALD, metabolic dysfunction-associated steatotic liver disease with increased alcohol intake; SD, standard deviation; SLD, steatotic liver disease.

**Table 2:** Associations of SLD status and CMRF count with aortic aneurysm and aortic dissection.

Group	Number	Events	Follow-Up Duration (Person-Years)	Incidence Rate (per 1000 Person-Years)	Crude HR (95% CIs, *p*-Value)	Adjusted HR (95% CIs, *p*-Value)
**SLD**						
**Aortic a** **neurysm**						
No SLD without CMRF	20504	42	196,613	0.21	1 (Reference)	1 (Reference)
No SLD with CMRF	125,258	498	1,191,963	0.42	1.95 (1.42–2.67, *p* < 0.001)	1.38 (1.00–1.89, *p* = 0.049)
MASLD	79,148	403	750,633	0.54	2.51 (1.83–3.45, *p* < 0.001)	1.58 (1.14–2.18, *p* = 0.005)
MetALD	11,342	51	106,975	0.48	2.24 (1.49–3.37, *p* < 0.001)	1.44 (0.95–2.18, *p* = 0.082)
ALD	3822	24	35,860	0.67	3.13 (1.90–5.17, *p* < 0.001)	1.71 (1.03–2.83, *p* = 0.038)
**Aortic dissection**						
No SLD without CMRF	20,504	8	196,773	0.04	1 (Reference)	1 (Reference)
No SLD with CMRF	125,258	148	1,193,626	0.12	3.04 (1.49–6.19, *p* = 0.002)	2.03 (0.99–4.15, *p* = 0.053)
MASLD	79,148	111	752,071	0.15	3.62 (1.77–7.43, *p* < 0.001)	2.12 (1.03–4.38, *p* = 0.042)
MetALD	11,342	13	107,167	0.12	2.99 (1.24–7.22, *p* = 0.015)	1.96 (0.81–4.79, *p* = 0.137)
ALD	3822	5	35,925	0.14	3.42 (1.12–10.46, *p* = 0.031)	1.93 (0.62–5.94, *p* = 0.254)
**CMRF**						
**A** **ortic a** **neurysm**						
No CMRF	20,528	42	196,824	0.21	1 (Reference)	1 (Reference)
any 1 CMRF	39,106	108	372,585	0.29	1.36 (0.95–1.94, *p* = 0.092)	1.11 (0.78–1.59, *p* = 0.554)
any 2 CMRF	51,108	188	486,154	0.39	1.81 (1.30–2.53, *p* < 0.001)	1.34 (0.96–1.88, *p* = 0.085)
any 3 CMRF	52,530	223	499,947	0.45	2.09 (1.50–2.90, *p* < 0.001)	1.45 (1.04–2.03, *p* = 0.028)
any 4 CMRF	48,423	274	459,523	0.60	2.79 (2.02–3.86, *p* < 0.001)	1.76 (1.27–2.45, *p* = 0.001)
all 5 CMRF	28,379	183	267,012	0.69	3.21 (2.30–4.49, *p* < 0.001)	1.76 (1.25–2.48, *p* = 0.001)
Per 1 CMRF					1.25 (1.20–1.31, *p* < 0.001)	1.13 (1.08–1.18, *p* < 0.001)
**Aortic dissection**						
No CMRF	20,528	8	196,824	0.04	1 (Reference)	1 (Reference)
any 1 CMRF	39,106	31	372,585	0.08	2.05 (0.94–4.45, *p* = 0.071)	1.67 (0.77–3.64, *p* = 0.195)
any 2 CMRF	51,108	64	486,154	0.13	3.23 (1.55–6.74, *p* = 0.002)	2.33 (1.11–4.87, *p* = 0.025)
any 3 CMRF	52,530	57	499,947	0.11	2.80 (1.33–5.86, *p* = 0.006)	1.82 (0.87–3.84, *p* = 0.114)
any 4 CMRF	48,423	75	459,523	0.16	4.00 (1.93–8.29, *p* < 0.001)	2.27 (1.08–4.74, *p* = 0.030)
all 5 CMRF	28,379	50	267,012	0.19	4.59 (2.18–9.68, *p* < 0.001)	2.21 (1.03–4.73, *p* = 0.041)
Per 1 CMRF					1.24 (1.14–1.34, *p* < 0.001)	1.08 (0.99–1.18, *p* = 0.079)

The model was adjusted for age, sex, income level, residence, Charlson comorbidity index, hemoglobin level, glomerular filtration rate, smoking status and physical activity. CI, confidence interval; CMRF, cardiometabolic risk factor; HR, hazard ratio; MASLD, metabolic dysfunction-associated steatotic liver disease; SLD, steatotic liver disease.

## Data Availability

Restrictions apply to the availability of these data. Data were obtained from the Korean National Health Insurance Service (NHIS) and may be accessed through the NHIS after approval. The authors are not permitted to share the data directly due to privacy and legal restrictions.

## Supplementary Materials

The following supporting information can be downloaded at https://www.mdpi.com/article/10.3390/jcm15145453/s1, Table S1: Baseline characteristics of the study population; Table S2: The operational definition of variables; Table S3: Baseline characteristics of the study population according to the number of cardiometabolic risk factors; Table S4: Sensitivity analysis using a Fatty Liver Index cutoff of 60 and the Hepatic Steatosis Index; Table S5: Associations of individual cardiometabolic risk factor components with the risks of aortic aneurysm and aortic dissection; Figure S1: Restricted cubic spline plots for the association between individual cardiometabolic risk factors and incident aortic aneurysm and dissection.

## Abbreviations

The following abbreviations are used in this manuscript:

AAA	abdominal aortic aneurysm
ALD	alcohol-associated liver disease
BMI	body mass index
CI	confidence interval
CMRF	cardiometabolic risk factor
FLI	Fatty Liver Index
HDL	high-density lipoprotein
HR	hazard ratio
HSI	Hepatic Steatosis Index
ICD-10	International Classification of Diseases, 10th Revision
LDL	low-density lipoprotein
MAFLD	metabolic dysfunction-associated fatty liver disease
MASLD	metabolic dysfunction-associated steatotic liver disease
MetALD	metabolic dysfunction-associated steatotic liver disease with increased alcohol intake
NAFLD	non-alcoholic fatty liver disease
NHIS	National Health Insurance Service
SD	standard deviation
SLD	steatotic liver disease
