# Supplementary material for: Association Between Metabolic Dysfunction-Associated Steatotic Liver Disease and Risk of Aortic Aneurysm and Dissection: A Nationwide Cohort Study
Source: J Clin Med. 2026 Jul 12;15(14):5453. doi: 10.3390/jcm15145453 (PMC13411413; doi:10.3390/jcm15145453)

Supplementary materials

Supplementary Table S1. All baseline characteristics of the study population

Supplementary Table S2. The operational definition of variables.

Supplementary Table S3. Baseline characteristics of study population according to the number of cardiometabolic risk factors.

Supplementary Table S4. Sensitivity analysis using a Fatty Liver Index cutoff of 60 and the Hepatic Steatosis Index.

Supplementary Table S5. Associations of individual cardiometabolic risk factor components with the risks of aortic aneurysm and aortic dissection.

Supplementary Figure S1. Restricted cubic spline plots for the association between individual cardiometabolic risk factors and incident aortic aneurysm and dissection.

Supplementary Table S1. All baseline characteristics of the study population.

Variables		No SLD without CMRF (n=20504)	No SLD with CMRF (n=125258)	MASLD (n=79148)	MetALD (n=11342)	ALD (n=3822)	P-value
Sex (%)	Male	9906 (48.3)	52908 (42.2)	53140 (67.1)	10864 (95.8)	3742 (97.9)	< 0.001
	Female	10598 (51.7)	72350 (57.8)	26008 (32.9)	478 (4.2)	80 (2.1)	
Age (years)	Mean (SD)	55.0 (7.2)	59.0 (8.9)	58.9 (8.5)	55.9 (7.2)	57.4 (8.2)	< 0.001
Income level (%)	1st quartile	2748 (13.4)	18526 (14.8)	10566 (13.3)	1268 (11.2)	489 (12.8)	< 0.001
	2nd quartile	4460 (21.8)	26962 (21.5)	15346 (19.4)	2067 (18.2)	765 (20.0)	
	3rd quartile	5610 (27.4)	36077 (28.8)	23463 (29.6)	3563 (31.4)	1263 (33.0)	
	4th quartile	7686 (37.5)	43693 (34.9)	29773 (37.6)	4444 (39.2)	1305 (34.1)	
Residence (%)	Rural	6248 (30.5)	43482 (34.7)	27785 (35.1)	3969 (35.0)	1580 (41.3)	< 0.001
	Urban	14256 (69.5)	81776 (65.3)	51363 (64.9)	7373 (65.0)	2242 (58.7)	
Hypertension (%)		0 (0.0)	53535 (42.7)	44565 (56.3)	6243 (55.0)	2176 (56.9)	< 0.001
Diabetes (%)		0 (0.0)	13107 (10.5)	15298 (19.3)	2221 (19.6)	783 (20.5)	< 0.001
Dyslipidemia (%)		0 (0.0)	43916 (35.1)	41284 (52.2)	4583 (40.4)	1429 (37.4)	< 0.001
Charlson comorbidity index (%)	0	13376 (65.2)	63059 (50.3)	36564 (46.2)	6091 (53.7)	1925 (50.4)	< 0.001
	1	5205 (25.4)	34368 (27.4)	21404 (27.0)	2985 (26.3)	1047 (27.4)	
	2	1529 (7.5)	15632 (12.5)	10819 (13.7)	1311 (11.6)	482 (12.6)	
	≥ 3	394 (1.9)	12199 (9.7)	10361 (13.1)	955 (8.4)	368 (9.6)	
Body mass index (kg/m²)	Mean (SD)	20.8 (1.5)	22.9 (2.2)	26.1 (2.5)	25.4 (2.5)	25.3 (2.6)	< 0.001
Waist circumference (cm)	Mean (SD)	73.2 (5.8)	78.3 (6.3)	88.2 (6.2)	88.0 (6.3)	88.1 (6.7)	< 0.001

Systolic blood pressure (mmHg)	Mean (SD)	111.7 (9.4)	124.5 (15.0)	128.7 (14.8)	130.4 (14.5)	131.1 (15.1)	< 0.001
Diastolic blood pressure (mmHg)	Mean (SD)	70.1 (7.3)	76.9 (9.8)	79.9 (9.8)	81.7 (9.7)	81.7 (9.8)	< 0.001
Fasting blood glucose (mg/dL)	Mean (SD)	87.8 (7.4)	98.4 (21.6)	105.7 (28.2)	108.5 (29.6)	109.7 (30.0)	< 0.001
Total cholesterol (mg/dL)	Mean (SD)	189.6 (25.5)	198.9 (36.8)	206.7 (38.5)	203.2 (36.8)	201.1 (37.9)	< 0.001
Triglyceride (mg/dL)	Mean (SD)	81.2 (28.3)	106.7 (48.5)	187.9 (96.4)	201.3 (116.6)	202.9 (121.4)	< 0.001
HDL cholesterol (mg/dL)	Mean (SD)	62.2 (19.1)	56.2 (23.1)	50.5 (25.6)	53.5 (20.0)	56.0 (29.7)	< 0.001
LDL cholesterol (mg/dL)	Mean (SD)	111.3 (24.2)	121.9 (35.8)	120.1 (39.4)	110.8 (41.5)	106.7 (43.0)	< 0.001
Aspartate aminotransferase (U/L)	Mean (SD)	23.3 (9.5)	23.6 (8.1)	27.6 (14.4)	31.4 (24.9)	34.3 (24.8)	< 0.001
Alanine aminotransferase (U/L)	Mean (SD)	18.8 (10.9)	20.3 (10.0)	29.4 (18.0)	31.3 (23.1)	32.5 (23.2)	< 0.001
gamma-glutamyl transpeptidase (U/L)	Mean (SD)	21.9 (17.0)	22.2 (14.2)	48.8 (50.7)	86.7 (89.4)	110.4 (119.6)	< 0.001
Hemoglobin (g/dL)	Mean (SD)	13.5 (1.4)	13.5 (1.4)	14.3 (1.4)	14.9 (1.2)	14.9 (1.3)	< 0.001
Glomerular filtration rate (mL/min/1.73 m^2)	Mean (SD)	81.8 (30.9)	79.0 (29.2)	77.0 (32.1)	80.7 (34.8)	81.4 (32.3)	< 0.001
Smoking (%)	Never	14133 (68.9)	92139 (73.6)	44758 (56.5)	2843 (25.1)	948 (24.8)	< 0.001
	Former	2730 (13.3)	17392 (13.9)	18113 (22.9)	3806 (33.6)	1224 (32.0)	
	Current smoker	3641 (17.8)	15727 (12.6)	16277 (20.6)	4693 (41.4)	1650 (43.2)	
Alcohol drinking (%)		7763 (37.9)	40106 (32.0)	35517 (44.9)	11342 (100.0)	3822 (100.0)	< 0.001
Amount of Alcohol	Mean (SD)	41.3 (95.9)	37.0 (96.8)	39.1 (57.1)	287.3 (70.1)	644.7 (262.2)	< 0.001

drinking (g/week)							
Physical activity (%)	No	13586 (66.4)	85298 (68.2)	51517 (65.2)	6719 (59.4)	2519 (66.0)	< 0.001
	1-2 times/week	4240 (20.7)	22758 (18.2)	17117 (21.7)	2819 (24.9)	742 (19.4)	
	3-4 times/week	1670 (8.2)	10325 (8.3)	6631 (8.4)	1183 (10.5)	296 (7.8)	
	5 times/week	966 (4.7)	6625 (5.3)	3738 (4.7)	599 (5.3)	258 (6.8)	
Fatty liver index	Mean (SD)	7.6 (5.5)	15.0 (7.7)	50.9 (15.8)	57.7 (17.7)	60.2 (18.1)	< 0.001

Supplementary Table S2. The operational definition of variables.

	ICD-10 codes	Definition
**Exclusion criteria**		
Viral hepatitis, autoimmune hepatitis, alcoholic liver disease, toxic liver disease, Wilson's disease, biliary cholangitis	B15, B16, B17, B18, B19, K70, K71, K743, K744, K745, K754, E830, E831	Admission ≥ 1 or outpatient clinic ≥ 1
Any cancer	C code	Admission ≥ 1 or outpatient clinic ≥ 1
Decompensated liver cirrhosis	R17, R18, K72, I850, I864, I983, K767	Admission ≥ 1 or outpatient clinic ≥ 1
**Outcomes**		
Aortic aneurysm	I711, I712, I713, I714, I715, I716, I718, I719	Admission ≥ 1 or outpatient clinic ≥ 2
Aortic dissection	I710	Admission ≥ 1 or outpatient clinic ≥ 2
**Hepatic steatosis assessment**		
Fatty liver index (FLI)	$FLI = \frac{1}{1+e^{-x}} \times 100$ where x = 0.953×ln(triglyceride, mg/dL) + 0.139×BMI (kg/m^2) + 0.718×ln(γ-GTP, U/L) + 0.053×waist circumference (cm) − 15.745	
Hepatic steatosis index (HSI)	HSI = 8×ALT/AST ratio + BMI (kg/m^2) (+2, if diabetes; +2, if female)	
**Cardiometabolic risk factors**		
Hypertension	I10, I11	Admission ≥ 1 or outpatient clinic ≥ 1 with any anti-hypertensive medication
Diabetes	E11, E12, E13, E14	Admission ≥ 1 or outpatient clinic ≥ 1 with any anti-diabetic medication
Dyslipidemia	E78	Admission ≥ 1 or outpatient clinic ≥ 1 with any lipid-lowering medication

Supplementary Table S3. Baseline characteristics of study population according to the number of cardiometabolic risk factors.

Variables		No CMRF	any 1 CMRF	any 2 CMRF	any 3 CMRF	any 4 CMRF	all 5 CMRF	P-value
**Sex (%)**	Male	9930 (48.4)	21175 (54.1)	28729 (56.2)	29292 (55.8)	25860 (53.4)	15574 (54.9)	<0.001
	Female	10598 (51.6)	17931 (45.9)	22379 (43.8)	23238 (44.2)	22563 (46.6)	12805 (45.1)	
**Age (years)**	Mean (SD)	55.0 (7.2)	56.7 (8.2)	57.9 (8.5)	58.6 (8.6)	60.0 (8.8)	61.3 (8.9)	<0.001
**Income level (%)**	1st quartile	2751 (13.4)	5355 (13.7)	7085 (13.9)	7223 (13.8)	7035 (14.5)	4148 (14.6)	<0.001
	2nd quartile	4467 (21.8)	8285 (21.2)	10632 (20.8)	10826 (20.6)	9734 (20.1)	5656 (19.9)	
	3rd quartile	5620 (27.4)	11144 (28.5)	14841 (29.0)	15479 (29.5)	14401 (29.7)	8491 (29.9)	
	4th quartile	7690 (37.5)	14322 (36.6)	18550 (36.3)	19002 (36.2)	17253 (35.6)	10084 (35.5)	
**Residence (%)**	Rural	6256 (30.5)	13298 (34.0)	17666 (34.6)	18480 (35.2)	17341 (35.8)	10023 (35.3)	<0.001
	Urban	14272 (69.5)	25808 (66.0)	33442 (65.4)	34050 (64.8)	31082 (64.2)	18356 (64.7)	
**Hypertension (%)**		0 (0.0)	7431 (19.0)	19190 (37.5)	23958 (45.6)	31834 (65.7)	24106 (84.9)	<0.001
**Diabetes (%)**		0 (0.0)	928 (2.4)	3148 (6.2)	6198 (11.8)	8336 (17.2)	12799 (45.1)	<0.001
**Dyslipidemia (%)**		0 (0.0)	0 (0.0)	6925 (13.5)	21588 (41.1)	36099 (74.5)	26600 (93.7)	<0.001
**Charlson comorbidity index (%)**	0	13391 (65.2)	24005 (61.4)	28592 (55.9)	26598 (50.6)	20235 (41.8)	8194 (28.9)	<0.001

Variables		No CMRF	any 1 CMRF	any 2 CMRF	any 3 CMRF	any 4 CMRF	all 5 CMRF	P-value
	1	5210 (25.4)	10122 (25.9)	13891 (27.2)	14557 (27.7)	13838 (28.6)	7391 (26.0)	
	2	1532 (7.5)	3477 (8.9)	5507 (10.8)	6608 (12.6)	7507 (15.5)	5142 (18.1)	
	≥ 3	395 (1.9)	1502 (3.8)	3118 (6.1)	4767 (9.1)	6843 (14.1)	7652 (27.0)	
**Body mass index (kg/m²)**	Mean (SD)	20.8 (1.5)	22.6 (2.4)	23.6 (2.6)	24.3 (2.7)	25.1 (2.6)	26.1 (2.5)	<0.001
**Waist circumference (cm)**	Mean (SD)	73.2 (5.8)	77.9 (7.1)	80.9 (7.6)	82.9 (7.6)	84.8 (7.3)	87.5 (7.0)	<0.001
**Systolic blood pressure (mmHg)**	Mean (SD)	111.7 (9.4)	119.4 (13.3)	124.7 (14.9)	126.5 (14.9)	130.0 (14.5)	133.1 (14.4)	<0.001
**Diastolic blood pressure (mmHg)**	Mean (SD)	70.1 (7.3)	74.5 (9.0)	77.5 (9.8)	78.4 (9.8)	80.2 (9.7)	81.4 (9.9)	<0.001
**Fasting blood glucose (mg/dL)**	Mean (SD)	87.8 (7.4)	92.1 (13.6)	96.2 (18.6)	100.9 (23.4)	104.5 (27.7)	121.9 (32.8)	<0.001
**Total cholesterol (mg/dL)**	Mean (SD)	189.6 (25.5)	191.4 (25.9)	197.2 (31.0)	205.2 (37.5)	209.3 (43.2)	206.4 (47.0)	<0.001
**Triglyceride (mg/dL)**	Mean (SD)	81.3 (28.3)	94.8 (39.9)	116.9 (60.2)	148.9 (80.2)	175.7 (96.7)	185.9 (110.8)	<0.001
**HDL cholesterol (mg/dL)**	Mean (SD)	62.2 (19.1)	59.2 (20.9)	56.5 (25.0)	53.0 (25.2)	50.8 (24.0)	49.9 (23.6)	<0.001
**LDL cholesterol (mg/dL)**	Mean (SD)	111.3 (24.2)	113.6 (25.0)	118.2 (30.8)	123.6 (38.4)	124.7 (44.5)	120.6 (46.7)	<0.001
**Aspartate aminotransferase (U/L)**	Mean (SD)	23.3 (9.5)	24.1 (9.6)	25.0 (13.0)	25.6 (12.8)	26.4 (13.3)	27.7 (14.3)	<0.001
**Alanine aminotransferase (U/L)**	Mean (SD)	18.8 (10.9)	20.7 (11.7)	22.6 (14.6)	24.5 (14.6)	26.3 (16.1)	28.9 (18.4)	<0.001
**gamma-glutamyl transpeptidase (U/L)**	Mean (SD)	22.1 (18.8)	28.0 (34.6)	32.8 (41.0)	37.7 (46.3)	40.8 (49.3)	46.2 (56.2)	<0.001

Variables		No CMRF	any 1 CMRF	any 2 CMRF	any 3 CMRF	any 4 CMRF	all 5 CMRF	P-value
**Hemoglobin (g/dL)**	Mean (SD)	13.5 (1.4)	13.7 (1.5)	13.9 (1.5)	14.0 (1.5)	14.0 (1.5)	14.0 (1.5)	<0.001
**Glomerular filtration rate (mL/min/1.73 m²)**	Mean (SD)	81.8 (30.9)	80.8 (31.0)	79.5 (30.4)	78.4 (29.6)	77.1 (30.4)	75.4 (32.6)	<0.001
**Smoking (%)**	Never	14138 (68.9)	25623 (65.5)	32700 (64.0)	33162 (63.1)	31018 (64.1)	18180 (64.1)	<0.001
	Former	2736 (13.3)	6407 (16.4)	9349 (18.3)	9883 (18.8)	9076 (18.7)	5814 (20.5)	
	Current smoker	3654 (17.8)	7076 (18.1)	9059 (17.7)	9485 (18.1)	8329 (17.2)	4385 (15.5)	
**Alcohol drinking (%)**		7787 (37.9)	16366 (41.9)	21916 (42.9)	22154 (42.2)	19139 (39.5)	11188 (39.4)	<0.001
**Amount of Alcohol drinking (g/week)**	Mean (SD)	42.0 (98.2)	55.9 (120.3)	62.2 (131.3)	64.3 (133.4)	60.1 (131.0)	62.6 (135.3)	<0.001
**Physical activity (%)**	No	13607 (66.4)	25632 (65.7)	33589 (65.8)	34968 (66.7)	32668 (67.6)	19175 (67.7)	<0.001
	1-2 times/week	4242 (20.7)	8107 (20.8)	10275 (20.1)	10594 (20.2)	9215 (19.1)	5243 (18.5)	
	3-4 times/week	1671 (8.2)	3234 (8.3)	4443 (8.7)	4239 (8.1)	4085 (8.4)	2433 (8.6)	
	5 times/week	966 (4.7)	2040 (5.2)	2705 (5.3)	2626 (5.0)	2376 (4.9)	1473 (5.2)	
**Fatty liver index**	Mean (SD)	7.7 (5.6)	15.5 (12.8)	23.6 (17.2)	32.2 (20.8)	40.0 (22.6)	47.5 (22.4)	<0.001

Supplementary Table S4. Sensitivity analysis using a Fatty Liver Index cutoff of 60 and the Hepatic Steatosis Index.

Group	Number	Events	Follow-up duration (person-years)	Incidence rate (per 1000 person-years)	Crude HR (95% CIs, P-value)	Adjusted HR (95% CIs, P-value)
**FLI ≥ 60**						
**Aneurysm**						
**No SLD without CMRF**	20252	42	196825	0.21	1 (Reference)	1 (Reference)
**No SLD with CMRF**	191311	841	1819435	0.46	2.16 (1.59-2.95, p<0.001)	1.45 (1.06-1.99, p=0.019)
**MASLD**	21486	102	202932	0.5	2.36 (1.65-3.38, p<0.001)	1.49 (1.04-2.15, p=0.031)
**MetALD**	4854	21	45839	0.46	2.15 (1.28-3.64, p=0.004)	1.41 (0.83-2.39, p=0.205)
**ALD**	1898	12	17768	0.68	3.17 (1.67-6.01, p<0.001)	1.79 (0.94-3.41, p=0.078)
**Dissection**						
**No SLD without CMRF**	20252	8	196957	0.04	1 (Reference)	1 (Reference)
**No SLD with CMRF**	191311	248	1821678	0.14	3.34 (1.65-6.76, p=0.001)	2.14 (1.05-4.34, p=0.036)
**MASLD**	21486	25	203217	0.12	3.03 (1.37-6.72, p=0.006)	1.74 (0.78-3.89, p=0.180)
**MetALD**	4854	3	45913	0.07	1.61 (0.43-6.08, p=0.480)	1.02 (0.27-3.89, p=0.972)
**ALD**	1898	1	17798	0.06	1.38 (0.17-11.05, p=0.760)	0.77 (0.10-6.20, p=0.807)
**HSI**						
**Aneurysm**						
**No SLD without CMRF**	20424	42	195847	0.21	1 (Reference)	1 (Reference)
**No SLD with CMRF**	170889	779	1618979	0.48	2.24 (1.64-3.06, p<0.001)	1.45 (1.06-1.98, p=0.021)

Group	Number	Events	Follow-up duration (person-years)	Incidence rate (per 1000 person-years)	Crude HR (95% CIs, P-value)	Adjusted HR (95% CIs, P-value)
**MASLD**	44385	179	426206	0.42	1.95 (1.39-2.73, p<0.001)	1.50 (1.06-2.11, p=0.021)
**MetALD**	3293	12	31424	0.38	1.78 (0.94-3.39, p=0.077)	1.33 (0.70-2.53, p=0.388)
**ALD**	979	6	9336	0.64	2.99 (1.27-7.04, p=0.012)	1.91 (0.81-4.51, p=0.139)
**Dissection**						
**No SLD without CMRF**	20424	8	195979	0.04	1 (Reference)	1 (Reference)
**No SLD with CMRF**	170889	215	1621039	0.13	3.24 (1.60-6.57, p=0.001)	2.05 (1.01-4.16, p=0.048)
**MASLD**	44385	57	426715	0.13	3.25 (1.55-6.82, p=0.002)	2.06 (0.97-4.37, p=0.059)
**MetALD**	3293	5	31467	0.16	3.90 (1.27-11.91, p=0.017)	2.75 (0.89-8.48, p=0.078)
**ALD**	979	0	9359	0	Not available	Not available

*The model was adjusted for age, sex, income level, residence, Charlson comorbidity index, hemoglobin level, glomerular filtration rate, smoking status and physical activity. CI, confidence interval; CMRF, cardiometabolic risk factor; HR, hazard ratio; MASLD, metabolic dysfunction-associated steatotic liver disease; SLD, steatotic liver disease

Supplementary Table S5. Associations of individual cardiometabolic risk factor components with the risks of aortic aneurysm and aortic dissection.

Group	Number	Events	Follow-up duration (person-years)	Incidence rate (per 1000 person-years)	Crude HR (95% CIs, P-value)	Adjusted HR (95% CIs, P-value)
**Aneurysm**						
Visceral obesity	156361	695	1493177	0.47	1.13 (0.99-1.29, p=0.061)	1.18 (1.03-1.35, p=0.019)
Insulin resistance	94456	434	890402	0.49	1.16 (1.03-1.32, p=0.016)	0.88 (0.77-1.00, p=0.044)
Hypertension	142825	815	1347372	0.6	2.79 (2.39-3.25, p<0.001)	1.71 (1.45-2.00, p<0.001)
High triglyceride	126288	620	1198527	0.52	1.41 (1.24-1.60, p<0.001)	1.18 (1.04-1.34, p=0.010)
Low HDL-cholesterol	114569	600	1090621	0.55	1.56 (1.38-1.77, p<0.001)	1.48 (1.30-1.69, p<0.001)
**Dissection**						
Visceral obesity	156361	204	1495098	0.14	1.33 (1.03-1.72, p=0.031)	1.23 (0.95-1.60, p=0.119)
Insulin resistance	94456	115	891629	0.13	1.06 (0.84-1.34, p=0.632)	0.80 (0.63-1.03, p=0.081)
Hypertension	142825	230	1349587	0.17	2.90 (2.16-3.89, p<0.001)	1.85 (1.36-2.51, p<0.001)
High triglyceride	126288	174	1200231	0.14	1.42 (1.12-1.80, p=0.004)	1.12 (0.88-1.43, p=0.346)
Low HDL-cholesterol	114569	157	1092344	0.14	1.33 (1.06-1.69, p=0.015)	1.09 (0.86-1.39, p=0.480)

*The model was adjusted for age, sex, income level, residence, Charlson comorbidity index, hemoglobin level, glomerular filtration rate, smoking status and physical activity.

Supplementary Figure S1. Restricted cubic spline plots for the association between individual cardiometabolic risk factors and incident aortic aneurysm and dissection.

*The model was adjusted for age, sex, income level, residence, Charlson comorbidity index, hemoglobin level, glomerular filtration rate, smoking status and physical activity. HDL-C, high-density lipoprotein cholesterol.

1) Aortic aneurysm

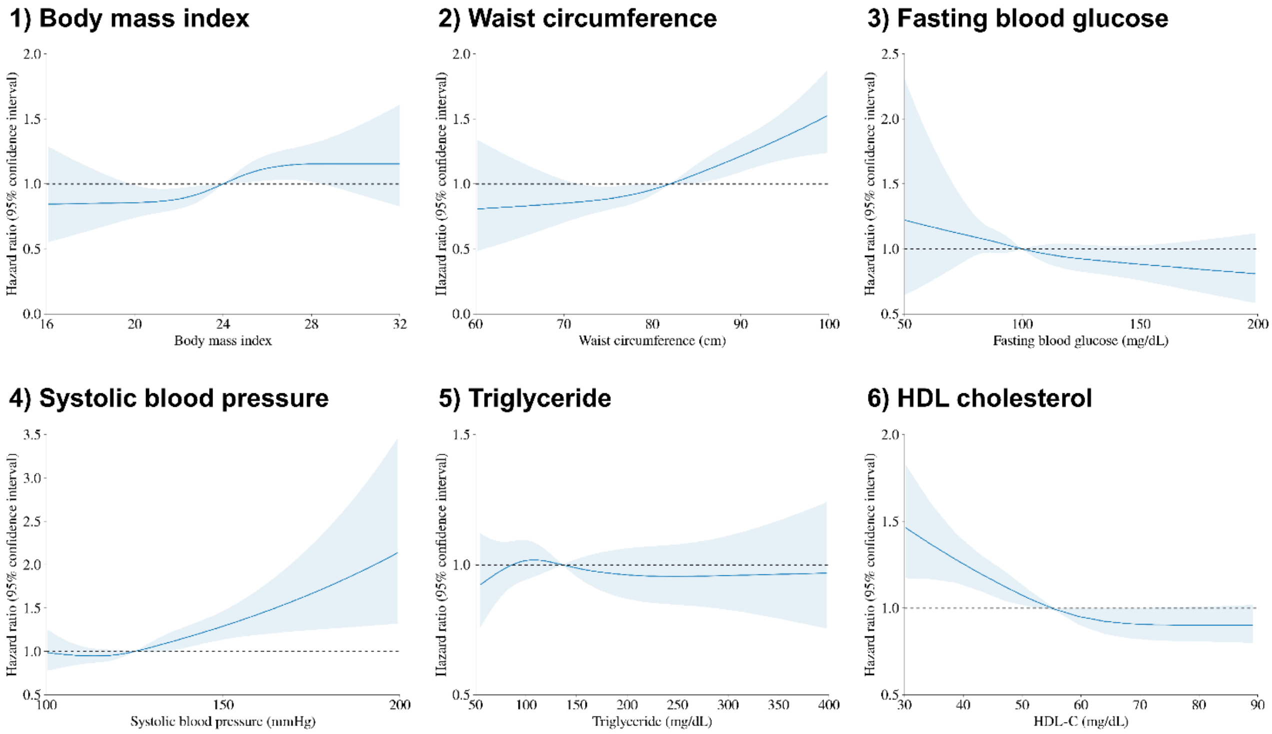

2) Aortic dissection

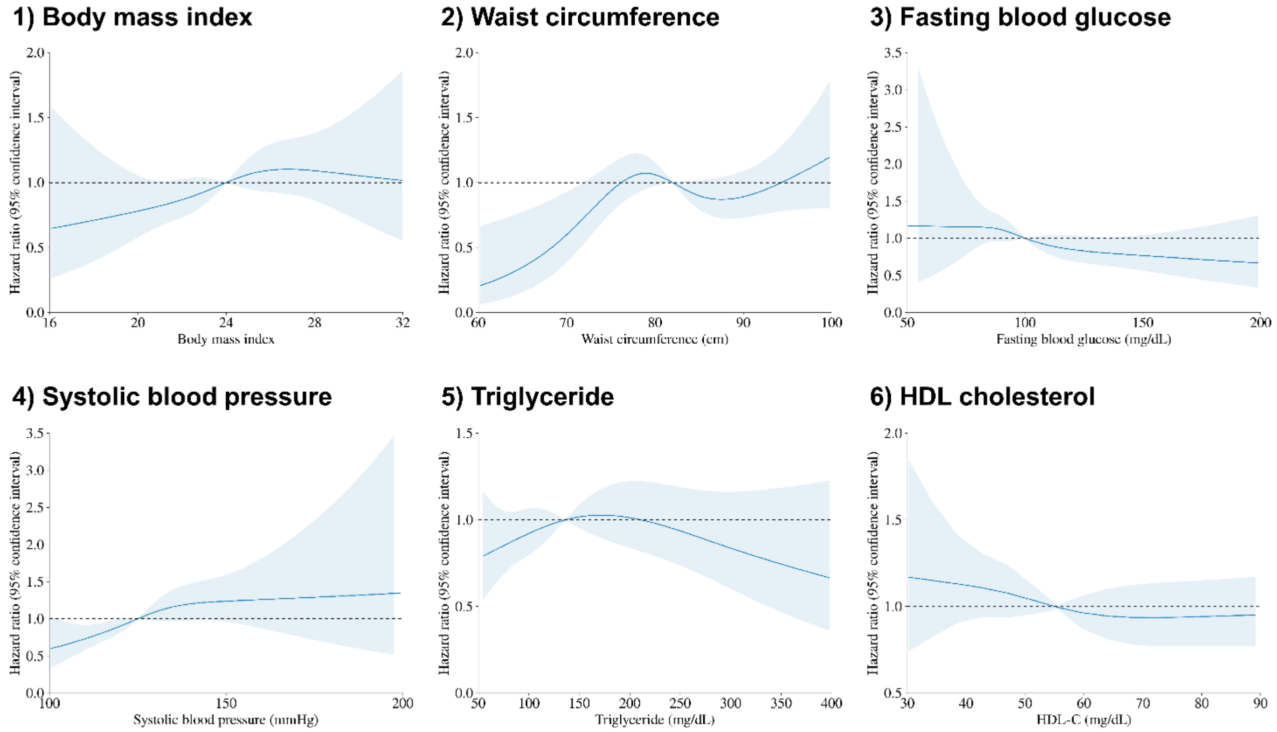

Supplement: Supplementary file 1 [file jcm-15-05453-s001.zip › jcm-4405579-supplementary.pdf]
